# **Comparison of Tympanoplasty Results in** **Dry and Wet Ears**

**Published:** 2016-05

**Authors:** Masoud Naderpour, Nikzad Shahidi, Taghi Hemmatjoo

**Affiliations:** 1 *Department of Otorhinolaryngology Head and Neck Surgery, Imam Reza Hospital, Tabriz, Iran.*

**Keywords:** Chronic Otitis Media, Dry Ear, Tympanoplasty, Wet Ear

## Abstract

**Introduction:**

Tympanoplasty is the standard and well established procedure for closure of tympanic membrane perforations .This paper compares the results of tympanoplasty in terms of hearing improvement and graft incorporation in patients with chronic perforation of the tympanic membrane between two groups with and without active drainage at the time of surgery.

**Materials and Methods:**

Sixty referring patients to specialty and subspecialty clinics between the age 15 to 60 years-old were selected. All patients suffered from Chronic Otitis Media and they were categorized into two groups: a) those with wet ears and b) those with dry ears. Tympanoplasty surgery was performed through the use of embedding technique of temporalis fascia graft and in medial position (Medial Graft Technique). Finally, the data about the level of hearing improvement and the repair of tympanic membrane were analyzed.

**Results:**

Although there was hearing improvement in both groups - with wet or dry ear - no statistically significant difference was observed between two groups. Following the surgery, tympanic membrane in two patients with wet ear and one with dry ear was not repaired, however according to the statistical analysis this difference was not significant.

**Conclusion:**

The results of this study showed that in contrast to the common perception that tympanoplasty results in the patients with wet ear is poorer than those with dry ear, there was little difference in the results of the operations performed on two groups.

## Introduction

Patients with chronic otitis media who suffer from ear discharge and hearing loss consist of a large percentage of the patients referred to subspecialty ENT clinics. Usually, surgeons perform tympanoplasty on ears with active drainage after drying the ear, but in many cases this is practically impossible because the discharge from the ear continues despite receiving medical treatment (1). This makes the decision of the date of operation quite hard. Given that no serious studies have yet examined the outcomes and outputs of tympanoplasty surgery in patients with persistent mucoid discharge, this study tries to reflect the results gained in the tympanoplasty surgery performed on patients with chronic perforation of the tympanic membrane. The size of the tympanic membrane perforation of these patients varied from small to subtotal and the surgeries were performed on both dry ears (no ear discharge at the time of surgery) and wet ears (consistent ear discharge at the time of surgery). In addition, tympanoplasty was performed through the postauricular approach and with the use of a temporal fascia graft and medial graft placement (underlay). The objectives of the study include: determining the hearing status before and after tympanoplasty surgery, evaluating the status of the tympanic membrane after the surgery, comparing the surgery results in terms of hearing improvement, and graft uptake rates in the patients with chronic perforation of the tympanic membrane with and without active discharge.

## Materials and Methods

Inclusion criteria in the study were:

1. Age between 15 to 60 years 

2. Having an infection background of Chronic Otitis Media and perforated tympanic membrane.

3. Lack of acute upper respiratory tract infection (URTI) at the time of surgery

Exclusion criteria in the study were:

1. Underlying diseases such as diabetes or poor immune system.

2. Definite diagnosis of Cholesteatoma and ossicular erosion in the selected patients3. Acute Otitis Media.

4. Presence of a Sensorineural Hearing Loss (SNHL). 

5. Definite diagnosis of Tympanosclerosis because such ears were frozen and could cause bias in results.

In this study, a total of sixty referring patients were selected from February 2010 to February 2014. All patients demonstrated a history of chronic otitis media and a perforated tympanic membrane. All cases were selected considering the inclusion criteria to the study and were treated with local and systemic antibiotics. Those with recurrence after 2 weeks were considered to suffer from a persistent infection. All patients were examined by microscope and a pure tone audiometry (PTA) test was performed. All selected patients for surgery were examined and the routine laboratory tests were applied. Afterwards, the subjects with diabetes, acute respiratory infection, weak immune system, and sensorineural hearing loss were excluded. 

The method of surgery (Tympanoplasty), used in all patients, was the same. Surgeries were performed through a postauricular approach and application of medial graft technic (underlay). Mastoidectomy was performed only in cases with polypoid middle ear mucosa or presence of polypoid tissue around the ossicles. The surgical team for all patients was the same and all the surgeries were performed in the operating room of Imam Reza Hospital. The patients suffering from Cholesteatoma, ossicular erosion and tympanosclerosis were also excluded from the study during surgery. 

Moreover, ossicular movement was controlled in all patients. In the case of its presence, hyaline membranes were removed from the bones and the bones were moved afterward. 

All patients were visited one day after the surgery. If there was no problem (postoperative complications), patients were discharged from the hospital. Regarding postoperative care, all patients were instructed and additionally received the needed notes and orders in a written form. 

All patients were followed up for the first time one week after the surgery and discharge. Then, their ears bandages were removed, and the status of the surgical site was examined. Except a slight inflammation at the surgical site, no complications such as hematoma, abscess or wound infection were observed. Recommendations regarding postoperative care were emphasized again. 

Patients were also followed up two other times after four and six weeks postoperatively. During these visits a microscopic examination of the ears was performed and the status of their postoperative graft was recorded. 

All patients underwent a PTA test in the 6th week for early detecting of graft taking and air bone gap (ABG) was calculated for all the patients. Closed ABGs were considered as 10 dB.

The final follow up was carried out 3 months after the surgery and microscopic examinations were performed, along with a PTA test to determine the final state of graft. The results showed no considerable change compared to the results of the 6th week. 

Data were analyzed by SPSS 16 software. To compare the aural status before and after the surgery in each group, a paired t test was applied. Finally, in order to check the hearing improvement rate in two groups, a t test was also used. 

## Results

This study was conducted on a total of 60 patients with chronic otitis media. The patients were categorized into two groups, those with wet ears and those with dry ears. 30 patients had dry ears and 30 of them had wet ears. 33 patients (55%) were females and 27 (45%) were males. 

The number of male patients with wet ears was 14 (46.66%) and that of the female patients was 16 (53.33%). Meanwhile, among the patients with dry ears, 13 (43.33%) were male and 17 (56.66%) were female. There was no statistically significant difference between the two groups in terms of gender (P= 0.5).

The mean age of the patients with wet ears was 32 (SD=10.8) and 33.9 (SD=9.96) for patients with dry ears. There was also no statistically significant difference between the two groups in terms of age (P=0.385).

Amongst the 60 patients involved in the study, 34 (56%) had a central perforation of tympanic membranes and 26 (43%) had subtotal perforation. Furthermore, there was no case with total or marginal perforation of tympanic membranes. 

From the 30 people with wet ears, 16 (53%) had central perforation and 14 (46%) had medium to subtotal perforation. In contrast, among the patients with dry ears 18 (60%) had central perforation and 12 (40%) had medium to subtotal perforation.

All 60 patients underwent tympanoplasty and in 5 patients (8%) simultaneous mastoidectomy was also performed. All of these five patients were from the group with wet ears.

Among the 60 patients, ossicular fixation was observed during the surgery in 2 patients (3%). One of these patients had dry ears while the other had wet ears (1.5%).

*Hearing level before and after the surgery on dry ears:*

Based on air bone gap (measured by PTA), moderate hearing loss in patients with dry ears was 40.36 dB (SD= 8.91) before the surgery, but this number reached 13 dB (SD=8.51) after the surgery. There was a statistically significant difference between hearing levels observed before and after the surgery in dry ears (P= 0.002).

*Hearing level before and after the surgery on wet ears:*

Based on air bone gap (measured by PTA), moderate hearing loss in patients with wet ears was 41.5 dB (SD=10.09) before the surgery, but this number reached 16.33 dB (SD=10.90) after the surgery. There was also a statistically significant difference between hearing levels observed before and after the surgery in wet ears (P= 0.001).

*Comparison of Hearing improvement in both groups:*

Based on air bone gap (measured by PTA), the average hearing improvement in all dry ears patients before and after the surgery was 26.2 dB (SD=8.31), while this number was 25.16 dB (SD=9.86) for those with wet ears. There was also no statistically significant difference between hearing improvements in dry and wet ears (P=0.583).

In 3 patients with dry ears, ABG was more than 20 dB. One of these patients had ossicular fixation and there was also one more case with Otorrhea and postoperative perforated membrane. 

In patients with wet ears, ABG was over 20 dB in 5 cases. One of these patients had ossicular fixation and there were also two more cases with otorrhea and postoperative perforated membranes. The remaining patients had the ABG of 20 dB or less.

*Restoration Results and type of ear discharge:*

Through microscopic examination after the surgery it was observed that graft incorporation had failed in one dry ear case (3%) and 2 wet ear cases (6%). Tympanic membrane perforation and Otorrhea were observable in these cases. However, tympanic membranes of the rest of patients had been repaired, namely 28 cases out of 30 patients with wet ears (93.3%) and 29 out of 30 patients with dry ears (96.7%) had successful operations in terms of closing the membrane perforation. However, according to the analysis it was proved that there was no significant difference between the status of dry the tympanic membrane and that of the wet one (Table. 1).

**Table 1:** Graft incorporation in both groups

	**Number of Patients**	**Postoperative Healthy Membrane**	**Percentage Success)**	
Status of Ear Discharge at the Time of Surgery	Wet	30	28	93.33%
	Dry	30	29	96.7%
	Total	60	57	95%

## Discussion

Tympanoplasty is an operation that removes infection and restore middle ear function in the ears with chronic otitis media. This operation is performed through surgical techniques and uses the materials which the surgeon chooses for the tympanic membrane graft. There have been a number of investigations regarding the impact of various factors such as age, sex, perforation size, the status of the opposite ear, discharge status of the ear at the time of surgery, surgical approaches and techniques, and the materials used for the graft. Based on these studies, most centers mainly use the approaches and techniques preferred by their own surgeons (1).

Robert and colleagues examined the risk factors of re-perforation following Myringoplasty surgery. They analyzed the impact of such factors such as age, sex, ear discharge, status of the opposite ear, hearing loss and surgical approach and technique on the results of Myringoplasty. Based on a statistical analysis, they finally introduced surgical technique as the more effective factor on the final results of Myringoplasty (2). 

Similarly, in our study such factors as age, sex, and ear discharge had no impact on the final results of the surgery.

Ashfaque and colleagues examined the results of type 1 tympanoplasty with internal graft technique (underlay) in 100 dry-eared patients. They concluded that in 81% of the patients, tympanic perforation was initially closed following the surgery and hearing improvement was quite remarkable. But such factors such as age, sex, and the size of the perforation had no significant effect on the results of surgery (3).

In our study, the graft incorporation rate in dry-eared patients was higher (96.7%) than the aforementioned study, and the factors like age and sex also had no significant effect on the results of the surgery.

Assuming that tympanoplasty in completely dry and atrophic ears with central perforations is more likely to fail in comparison to wet ears with central perforations, Vijendra and colleagues (2007) performed histopathological examinations on the remaining tympanic membranes of the patients. They observed that in completely dry and atrophic membranes blood vessels are quite marginalized, while the membranes were either absent or as small as possible. In contrast, there were lots of inflammatory cells and blood vessels in the remaining membranes of wet ears. Therefore, they concluded that these types of changes in blood vessels are the main causes of failure in completely dry and atrophic membranes with central perforations. Hence, they recommended taking the following steps while operating on these types of ears and membranes: resection of the margins of perforations and converting central perforations to subtotal; raising large tympanomeatal flaps; temporal fascia graft placement between the bony wall of the canal and the large bloody flap. They believe that these measurements increase the chance of a successful operation. Contrary to Vijendra's results, the graft incorporation rate in dry eared patients was better than that of wet eared ones (96.7% compared to 93.3%). However, this difference was not statistically significant (4).

Nagle and colleagues examined the results of type 1 tympanoplasty in 100 wet eared and dry eared patients with perforated tympanic membrane. They also compared the aural status and closing of the membrane perforation in the two groups. 

The results showed that in 88% of dry eared patients and 74% of wet eared patients the perforated membrane had been closed following the surgery. Though, this difference was not statistically significant. There was also a significant improvement in the hearing status of patients in both groups, though there was no significant difference between the two groups in this respect (5).

The results of our study were in correlation with Nagle's study, and there was only a small difference in graft incorporation rates. The rates of our study in both wet eared and dry eared patients were better than those of Nagle's study.

Hosney and colleagues studied the outcome of myringoplasty in wet and dry ears and found that there was no statistically significant difference between wet and dry ears in graft taking and hearing improvement (6).

## Conclusion

In our study, tympanoplasty surgery results of both wet eared and dry eared groups were quite significant and satisfying in terms of both hearing improvement and graft incorporation. There was also no statistically significant difference between the two groups. Given these results, we might conclude that if an appropriate surgical technique is taken, and an efficient postoperative education is provided, then satisfying results can be achieved from operations on wet ears (just like dry ears). However, in order to be sure we need to carry out more studies with larger samples and in multiple centers where there are more variables to consider.
